# Gender differences in cardiometabolic health and disease in a cross-sectional observational obesity study

**DOI:** 10.1186/s13293-022-00416-4

**Published:** 2022-03-04

**Authors:** Christina Strack, Gundula Behrens, Sabine Sag, Margareta Mohr, Judith Zeller, Claas Lahmann, Ute Hubauer, Thomas Loew, Lars Maier, Marcus Fischer, Andrea Baessler

**Affiliations:** 1 Clinic of Internal Medicine II, University Hospital of Regensburg, Franz-Josef-Strauss-Allee 11, 93053 Regensburg, Germany; 2 Department of Epidemiology and Preventive Medicine, University of Regensburg, Franz-Josef-Strauss-Allee 11, 93053 Regensburg, Germany; 3 Department of Psychosomatics, University Hospital of Regensburg, Franz-Josef-Strauss-Allee 11, 93053 Regensburg, Germany; 4 Department of Psychosomatic Medicine and Psychotherapy, Faculty of Medicine, University Hospital of Freiburg, Hauptstrasse 8, 79104 Freiburg, Germany

**Keywords:** Sex differences, Cardiometabolic health, Metabolic syndrome, Body fat distribution, Adiponectin

## Abstract

**Background:**

Beyond the degree of adiposity, the pattern of fat distribution has a profound influence on cardiometabolic risk. It is unclear if sex differences in body fat distribution can potentially explain any sex differences in the prevalence of the metabolic syndrome (MetS) and in individual cardiometabolic risk factors among obese men and women.

**Methods:**

In this cross-sectional analysis, 432 persons from the ongoing Obesity Weight Reduction Study (*n* = 356 obese, ØBMI 41 ± 8 kg/m^2^, and 76 non-obese, ØBMI 25 ± 3 kg/m^2^), were included. The relations of sex to MetS prevalence and selected cardiometabolic risk factors were assessed using univariate and multivariate adjusted regression models.

**Results:**

In crude analyses, %fat mass and the fat mass/lean mass ratio were significantly higher in women than in men, regardless of increasing obesity categories, from normal weight to grade-3-obesity. In contrast, markers of abdominal obesity, such as waist circumference and waist-to-hip ratio were higher in men than in women, despite similar BMI. The prevalence of the MetS was higher in obese men than in women (67.6 vs. 45.0%, *p* < 0.0001), particularly in younger individuals < 40 years (72.5 vs. 36.8%, *p* < 0.0001), but “metabolically healthy obesity” (BMI ≥ 30, no other NCEP ATPIII MetS component) was more common in women than in men (15.6 vs. 4.1%, *p* < 0.0001). After adjusting for age, %body fat and height, sex differences were observed for HDL-cholesterol (*p* < 0.001), triglycerides (*p* < 0.001), fasting glucose (*p* = 0.002), insulin and HOMA-IR levels (*p* < 0.001), ALAT (*p* < 0.001), adiponectin (*p* < 0.001), and sE-selectin (*p* = 0.005). In contrast, crude sex differences in other variables, such as leptin levels (68 ± 4 in obese women vs. 33 ± 2 µg/L in men, *p* < 0.0001), disappeared after accounting for differences in %body fat (least-squares means of leptin: 52 ± 4 vs. 55 ± 6 µg /L, *p* = 0.740). A logistic regression model adjusting for age and lifestyle factors revealed a lower risk of having MetS for women as compared to men (OR = 0.38[0.22–0.60]). That risk estimate did not materially alter after adding BMI to the model. In contrast, no statistically significant association between sex and MetS prevalence was observed after adding waist circumference and adiponectin to the model (OR = 1.41[0.59–3.36]).

**Conclusions:**

Different body fat distribution patterns, particularly abdominal adiposity, adiponectin, and related biomarkers, may contribute to sex differences in cardiometabolic risk factors and to the prevalence of the MetS.

**Supplementary Information:**

The online version contains supplementary material available at 10.1186/s13293-022-00416-4.

## Introduction

Abdominal obesity is strongly associated with traditional cardiovascular risk factors clustered in the metabolic syndrome (MetS), such as abnormalities in glucose metabolism, dyslipidemia, as well as arterial hypertension, and is thus associated with an elevated risk of cardiovascular disease [1, 2].

The pathogenic effect of adipose tissue seems to be attributed in part to different body fat distribution patterns. In contrast to subcutaneous fat, which mainly serves as an organ of energy homeostasis and storage, high liver fat content and ectopic intra-abdominal fat are strongly associated with a metabolic unhealthy condition [3, 4]. Indeed, visceral fat accumulation is considered to be a key factor for obesity-related diseases, because its adipocytes secrete adverse adipokines that participate in a disturbed regulation of glucose and lipid metabolism, energy homeostasis and insulin sensitivity, inflammation, as well as vascular function and coagulation [5]. On the other hand, some obese individuals seem to be protected from obesity related cardiovascular risk factors, possibly due to the co-secretion of anti-inflammatory adipokines, such as adiponectin [3].

Women generally have a higher percentage of body fat and a lower percentage of lean mass than men. However, the prevalence of MetS is lower in women than in men of similar age even after controlling for body mass index (BMI) [6]. If a distinctive body fat distribution pattern with accumulation of abnormal adipose tissue cells especially in the visceral compartments is essential for the development of obesity related diseases, sex differences in body composition should result in differences in the prevalence of metabolic and cardiovascular alterations associated with obesity. This is because women have more favorable subcutaneous adipose tissue, especially in the lower body and the gluteal–femoral region, i.e., a female pear shaped body fat distribution, and men have predominantly unfavorable fat distributed to the visceral region around the abdominal organs [7, 8].

Although sex differences in fat distribution and correlations to metabolic health are somewhat established in the clinical and epidemiological literature, the biological underpinnings of these associations remain poorly understood, particularly in men and women with more severe obesity.

Previous studies in this field investigated sex differences of single aspects related to obesity, body composition, insulin resistance, or diabetes [9, 10], restricted their investigations to specific populations, e.g., the elderly (i.e., including postmenopausal women only) [11–13] or aimed to identify sex- and age-specific risk factors for the MetS [14] with or without consideration of body mass index [6]. Other investigations elucidated the potential role of sex hormones on body composition or metabolic issues, e.g., insulin resistance [9, 15] or liver function [10]. However, since several of these parameters are likely influenced by heterogeneities in extent, morphology and composition of adipose tissue (which are fundamentally different in men and women), it is still unknown whether acknowledged sex differences in these phenotypes are really sex-different or just a result of the given natural differences in body composition in men and women, i.e., higher adiponectin levels in women because of higher amount of subcutaneous fat tissue. Thus, it is crucial to elucidate the impact of the given natural sex differences in body composition on sex differences in MetS components.

Moreover, aging, hormonal influences, and environmental lifestyle factors such as alcohol consumption, physical exercise and food preferences additionally contribute in part to differences in body fat distribution patterns as well as adipose tissue health [16, 17].

Thus, to describe sex differences more thoroughly the present study aims to comprehensively investigate the association between sex, MetS components, metabolic, heart, liver and vascular health in predominantly very obese patients by taking into account natural sex differences in body composition and distribution, age, and lifestyle factors.

## Methods

### Study participants

For the present cross-sectional study, we used the baseline data from the prospective ‘Obesity Weight Reduction Study’ initiated in 2005, conducted at the University Hospital Regensburg, Germany. The study description was described earlier [18]. In brief, 432 subjects (173 men and 259 women) aged 18–69 years gave written informed consent to participate in our study. All study participants were Caucasians. All obese subjects (BMI ≥ 30 kg/m^2^, *n* = 356) intended to conduct a standardized weight reduction program. Non-obese individuals (*n* = 76) of similar age distributions were enrolled as controls. Participants were only enrolled if they had maintained a constant body weight during the last 3 months prior to enrollment and if they had not lost more than 10 percent of their weight during the last 6 months. The study was approved by the local ethics committee.

### Assessment of cardiometabolic risk factors at baseline

At the baseline investigation and interview, participants provided information on any chronic diseases diagnosed prior to enrollment including hypertension, dyslipidemia, type 1 and type 2 diabetes mellitus. In addition, participants reported on their smoking behaviour (current cigarette smoking intensity, age at which they had started or quitted smoking), alcohol, fruit and vegetable intakes (never, occasionally, 1–3 times per week, daily), and regular moderate to vigorous physical activity (defined as ≥ 3 times per week for ≥ 30 min). The baseline physical examinations included anthropometric measurements (height, weight, waist and hip circumference), bioelectrical impedance analysis (BIA; Nutriguard©-Impedance Analysis Apparatus, Data Input GmbH Darmstadt, Germany), blood pressure measurements, and echocardiography (Philips iE33 Philips Medical Systems, Hamburg, Germany). In addition, blood samples were collected at baseline.

### Definition of the metabolic syndrome

We used the National Cholesterol Education Program–Adult Treatment Panel III (NCEP ATP III) to define the MetS as meeting at least 3 of the following 5 criteria: 1. Waist circumference ≥ 88 cm in women and ≥ 102 cm in men; 2. Serum triglycerides ≥ 150 mg/dL; 3. Serum high-density lipoprotein cholesterol (HDL-C) ≤ 50 mg/dL in women and ≤ 40 mg/dL in men; 4. Systolic blood pressure ≥ 130 mmHg or diastolic blood pressure ≥ 85 mmHg or diagnosis of hypertension; 5. Fasting serum glucose ≥ 110 mg/dL or diagnosis of type 2 diabetes mellitus [19].

### Selection of the cardiometabolic health components

Metabolically healthy obesity was defined as the sole presence of obesity (BMI ≥ 30 kg/m^2^); none of the other NCEP ATP III MetS criteria had to be present.

We selected additional secondary outcome variables a priori. All selected outcome variables have been previously linked to the MetS. In particular, we examined insulin resistance [using both, a low cutoff level HOMA-IR > 2.6 ([20, 21] and a higher cutoff level HOMA-IR > 3.8 [22–24]], as well as a normal left ventricular diastolic heart function (defined according to the American Society of Echocardiography ASE/EAE algorithm as a septal pulsed-wave TDI e−velocity > 8 cm/sec combined with normal left atrial dimensions) [25].

### Selection of the potential confounding factors

We selected age, smoking, abdominal adiposity, general obesity, physical activity, and intakes of alcohol, fruit and vegetables as potential confounding factors a priori [6, 26–29].

### Statistical analysis

We compared continuously distributed baseline characteristics between women and men using one-way analysis of variance. Logistic regression analysis was used to examine the association of categorial independent variables with dichotomous variables. The Pearson *χ*^2^ test was used to assess for the independence of the rows and columns in standard two-way tables.

We calculated adjusted means and standard errors from linear regression estimates for one or two nominal X variables, adjusted for covariates.

Crude and adjusted means and their standard errors of blood parameters were compared using linear regression models in non-obese and obese study participants. Because our study included several adiposity measures, we used a hierarchical regression method of forward selection to decide which adiposity measures should be included in our model. Analyses were performed with the use of STATA software (StataCorp. 2015. *Stata Statistical Software: Release 14*. College Station, TX: StataCorp LP).

For each analysis the significance level a was set at 0.05 using two-sided statistical tests.

## Results

Characteristics of female and male study participants are displayed in Table 1. Mean age was comparable between female (44.0 ± 12.5) and male (45.5 ± 11.9 years, *p* = 0.230) subjects. Most obese study participants had severe obesity with grade 2 (BMI ≥ 35 kg/m^2^) or grade 3 obesity (BMI ≥ 40 kg/m^2^), i.e., in 69% of obese women and 78% of obese men (p = 0.172; n.s.). In total, female obese had a slightly lower mean BMI than male obese (40.0 ± 7.4 vs. 41.8 ± 8.2 kg/m^2^, *p* = 0.035). The MetS according to the NCEP ATP III criteria occurred in 95 women and 98 men. In the subset of obese individuals, the frequency of the MetS was significantly higher in men (67.6%) than in women (45.0%, *p* < 0.0001), particularly in younger individuals < 40 years of age (Table 1). Contrary, the frequency of metabolically healthy obesity (obesity with no other NCEP ATP III MetS component present) was significantly higher in women than in men (15.6 vs. 4.1%, *p* < 0.0001).

**Table 1:** Characteristics of female and male study participants

	Female	Male	*p* value
*n*	259	173	
Non-obese [n, (%)]	48 (18.5%)	28 (16.2%)	0.172*
Obese [n, (%)]	211 (81.5%)	145 (83.8%)	0.530
Age [years]	44.0 ± 12.5	45.5 ± 11.9	0.230
BMI			
Non-obese [kg/m^2^]	25.0 ± 3.6	24.1 ± 2.7	0.249
Obese [kg/m^2^]	40.0 ± 7.4	41.8 ± 8.2	0.035
Obesity grade [*n*, (% obese)]			
Grade 1 Obese [*n*, (%)]	66 (25.5%)	31 (17.9%)	0.172*
Grade 2 Obese [*n*, (%)]	51 (19.7%)	44 (25.4%)	0.172*
Grade 3 Obese [*n*, (%)]	94 (36.3%)	77 (40.5%)	0.172*
MetS [*n*, (% obese)]	95 (45.0%)	98 (67.6%)	< 0.0001
18–39 years [*n*, (%)]	28 (36.8%)	29 (72.5%)	< 0.0001
40–55 years [*n*, (%)]	48 (49.5%)	43 (67.2%)	0.037
> 55 years [*n*, (%)]	19 (50.0%)	26 (65.0%)	0.183
Metabolically Healthy Obese [n, (%)]	33 (15.6%)	6 (4.1%)	< 0.0001

*The Pearson χ^2^ test was used to calculate for the independence of the rows (sex) and columns (BMI categories) in a standard two-way table. The χ^2^ associated with this table has 3 degrees of freedom and is 5.0. The observed differences are non-significant

**Fig. 1:**
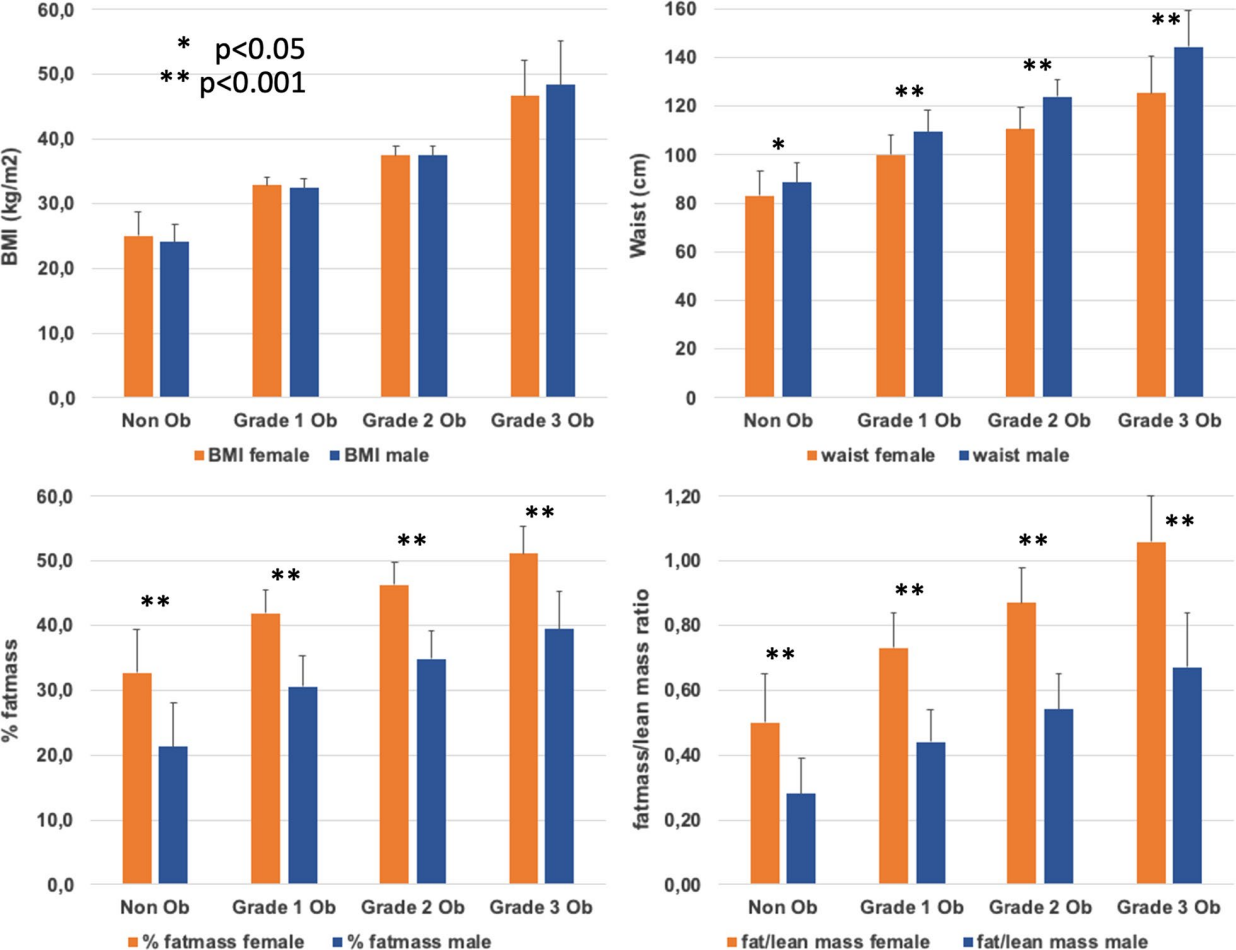
Sex differences in different body composition parameters in non-obese and obese study participants. Non Ob, non-obese with BMI < 30 kg/m^2^; Grade 1 Ob, obesity with BMI 30–35 kg/m^2^; Grade 2 Ob, obesity with BMI 35–40 kg/m^2^; Grade 3 Ob, obesity with BMI > 40 kg/m^2^

Different parameters of adiposity are depicted in Fig. 1. By definition BMI was comparable in men and women across increasing severity of obesity. In contrast, waist circumference, a marker of abdominal adiposity, also referred to as “visceral obesity”, was significantly higher in men than in women, regardless of the BMI group (Fig. 1). The same is true for waist/hip ratio (0.98 ± 0.09 vs. 0.85 ± 0.08, *p* < 0.0001) and epicardial fat thickness (6.8 ± 3.8 vs. 5.6 ± 3.1 cm, *p* = 0.0005), both being parameters of visceral obesity, too (data not shown). Nevertheless, %fat mass as well as fat mass/lean mass ratio was markedly higher in women than in men, in each class, from non-obese to grade 3 obesity. Thus, in our cohort of predominantly very obese men and women, a clear sex difference in body fat distribution is evident: women had lower indices of abdominal (visceral) obesity but a higher index of general obesity (here: percent body fat) across all obesity classes.

Lifestyle characteristics of study cohort stratified by sex and obesity status are given in Suppl. Table 1. In non-obese and obese study participants smoking behaviour and physical activity was similar in men and women. In contrast, alcohol consumption was significantly higher in men, but the consumption of fruits and vegetables was significantly lower in men than in women, particularly in the obese (Additional file 1: Table S1).

Several lipid parameters, parameters of insulin–glucose metabolism, liver enzymes, adipokines, and markers of inflammation and early atherogenesis were studied for sex differences across increasing obesity severity grades (Additional file 1: Table S2). Statistically significant differences between men and women could be observed for HDL cholesterol, triglycerides, ApoA1, fasting glucose and insulin levels, HOMA-IR, the liver enzymes ALAT and yGT, the adipokines leptin and adiponectin, high sensitive CRP, homocysteine, and the cell adhesion molecule sE-selectin. All those parameters were more favourable in women than in men apart from high sensitive CRP, which was higher in women than in men. Moreover, cardiovascular parameters, such as blood pressure parameters, intima media thickness, ankle/brachial index as well as echocardiographic parameters are shown in men and women in Additional file 1: Table S3. Obese men had less favourable cardiovascular values than women, such as higher systolic blood pressure and pulse pressure levels, a more accentuated intima media thickness, as well as more pathologic parameters of diastolic dysfunction. Of these, parameters which turned out to be statistically different between men and women in the initial univariate analysis, entered a more thoroughly investigation using multivariate adjustments, controlling for age and differences in body composition, such as height and percent fat mass (Table 2).

**Table 2:** Crude and adjusted means (adjusted for age, height, %fat mass) and their standard errors of blood parameters in non-obese and obese study participants, stratified by sex

	Non-obese female	Non-obese male	*p* value non-obese female vs. male	Obese female	Obese male	*p* value obese female vs. male
Lipids						
HDL-Chol [mg/dl]						
Crude	68 ± 3	59 ± 2	0.0274	54 ± 1	41 ± 1	< 0.0001
Adjusted*	72 ± 3	52 ± 5	0.0060	53 ± 1	42 ± 2	< 0.0001
Triglycerides [mg/dl]						
Crude	103 ± 9	103 ± 13	0.9992	129 ± 5	173 ± 7	< 0.0001
Adjusted*	76 ± 12	150 ± 18	0.0052	132 ± 8	170 ± 10	0.0190
Apo-A1 [mg/dl]						
Crude	185 ± 5	167 ± 4	0.0129	162 ± 2	140 ± 2	< 0.0001
Adjusted*	189 ± 6	160 ± 8	0.0212	160 ± 3	144 ± 4	0.0029
Glucose–insulin						
Glucose [mg/dl]						
Crude	85 ± 1	86 ± 2	0.7718	94 ± 1	110 ± 3	< 0.0001
Adjusted*	82 ± 2	91 ± 2	0.0166	93 ± 3	110 ± 3	0.0015
Insulin [mg/dl]						
Crude	8.8 ± 0.9	6.6 ± 0.7	0.0788	19.8 ± 1.3	28.7 ± 1.7	< 0.0001
Adjusted*	7.3 ± 1.0	9.4 ± 1.4	0.3200	16.1 ± 1.9	33.9 ± 2.5	< 0.0001
HOMA-IR [mg/dl]						
Crude	1.9 ± 0.2	1.4 ± 0.2	0.1181	4.9 ± 0.4	8.3 ± 0.7	< 0.0001
Adjusted*	1.5 ± 0.2	2.2 ± 0.3	0.1900	3.8 ± 0.6	9.5 ± 0.8	< 0.0001
***Liver***						
GPT(ALAT) [mg/dl]						
Crude	25 ± 2	30 ± 2	0.1124	31 ± 1	50 ± 3	< 0.0001
Adjusted*	21 ± 2	37 ± 3	0.0017	31 ± 2	51 ± 3	< 0.0001
γGT [mg/dl]						
Crude	26 ± 3	40 ± 12	0.1451	32 ± 2	54 ± 3	< 0.0001
Adjusted*	19 ± 8	51 ± 12	0.0644	32 ± 3	53 ± 3	0.0001
Adipokines						
Leptin [µg/L]						
Crude	19.4 ± 1.9	3.3 ± 0.3	< 0.0001	68.4 ± 4.2	32.8 ± 2.2	< 0.0001
Adjusted*	15.4 ± 1.8	11.2 ± 3.0	0.3200	52.0 ± 4.1	54.9 ± 5.6	0.7399
Adiponectin [µg/ml]						
Crude	12.2 ± 0.8	10.4 ± 1.8	0.2739	10.1 ± 0.3	7.1 ± 0.3	< 0.0001
Adjusted*	15.2 ± 1.2	5.4 ± 1.8	0.0004	9.9 ± 0.4	7.3 ± 0.5	0.0009
Hs-CRP [mg/L]						
Crude	1.7 ± 0.3	1.4 ± 0.4	0.5582	8.3 ± 0.7	5.5 ± 0.4	0.0016
Adjusted*	1.4 ± 0.4	2.0 ± 0.6	0.4717	6.1 ± 0.7	8.7 ± 1.0	0.0777
Homocystein [µmol/L]						
Crude	9.5 ± 0.3	11.0 ± 0.4	0.0059	10.1 ± 0.2	12.0 ± 0.5	0.0002
Adjusted*	9.1 ± 0.4	11.7 ± 0.7	0.0085	10.4 ± 0.5	11.6 ± 0.6	0.1961
SE-Selectin [ng/ml]						
Crude	32.7 ± 2.0	38.6 ± 2.6	0.0705	43.1 ± 1.8	49.7 ± 2.2	0.0221
Adjusted*	29.5 ± 2.6	44.0 ± 3.9	0.0119	40.3 ± 2.5	54.7 ± 3.4	0.0054

Using such multivariate analysis, statistically significant differences between men and women remained for HDL cholesterol, triglycerides, fasting glucose, insulin, and HOMA-IR levels, liver enzymes ALAT, yGT, adiponectin, and sE-selectin. In contrast, the quite considerable sex differences observed for crude leptin, hs-CRP, and homocysteine levels completely disappeared after accounting for age, height and %fat mass. These parameters seem to be strongly influenced by the relative amount of fat mass.

**Fig. 2:**
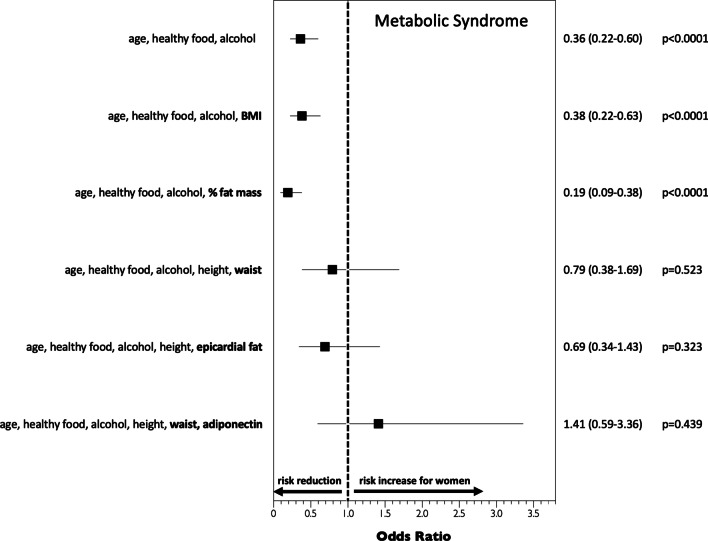
Different multivariate adjustment models analyzing the risk for having the MetS by sex

Using various statistical adjustment models the relative risk for the presence of the MetS by sex is depicted in Fig. 2. The apparent risk reduction in female obese patients is evident despite accounting for age, BMI, %body fat mass and lifestyle factors. However, when accounting for parameters of abdominal or visceral obesity (such as waist circumference or epicardial fat thickness), respectively, the risk became similar in men and women. In addition, accounting for adiponectin even led to a somewhat reversal of the risk with a tendency of a higher risk ratio in women compared to men, which was not statistically significant.

**Fig. 3:**
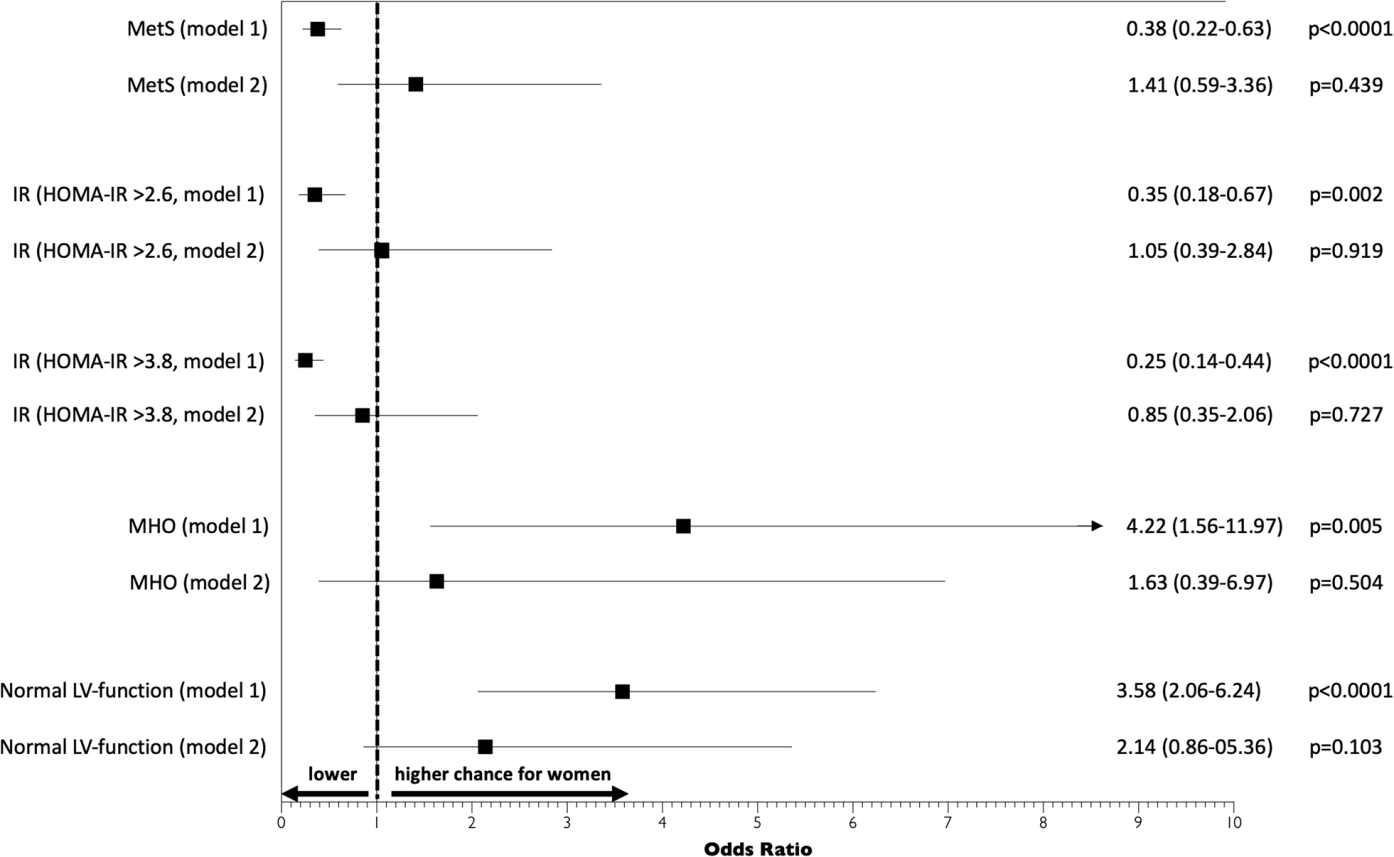
Odds ratios comparing the risk for cardiometabolic disturbances (MetS, insulin resistance) and health characteristics (MHO, metabolically healthy obesity, normal left ventricular function without evidence of systolic or diastolic dysfunction) in women vs. men using multivariate-adjusted logistic regression models. Model 1 adjusted for age, lifestyle factors (fruits- and vegetable consumption, alcohol intake) and body size (BMI). Model 2 adjusted for age, lifestyle factors (fruits- and vegetable consumption, alcohol intake) and visceral obesity parameters (waist, adiponectin, height)

Besides the risk ratios for the MetS, the risk ratios for related cardiometabolic disturbance such as insulin resistance and health characteristics such metabolically healthy obesity and normal left ventricular function is shown in Fig. 3 using statistical adjustment models with and without visceral obesity parameters (waist circumference) and adiponectin levels. The increased risks for the MetS as well as insulin resistance parameters for obese men observed in model 1 are equalized by additionally accounting for waist circumference and adiponectin levels (model 2). Analogously, the decreased chance of being metabolically healthy obese or having a normal left ventricular function for men (model 1) was also equalized by accounting for waist circumference and adiponectin levels (model 2, Fig. 3).

## Discussion

The underlying mechanisms for the obvious sex dimorphism of cardiometabolic disturbances are still not fully understood. To explore why obese men were more frequently affected by the metabolic syndrome (MetS) than obese women we analysed which cardiometabolic risk factors were different between obese men and women.

In the present investigation the association of male gender with the MetS could be confirmed despite a significantly higher body fat percentage in women in each WHO obesity grade.

We analysed the association of various cardiometabolic risk factors with sex both without and with consideration of potential confounding factors, being per se different between men and women. Specifically, it is fact that women in general have higher %body fat than men, and because adipose tissue produces or influences several cardiometabolic parameters by secreting hormones and adipokines, this could be one contributor for some of the apparent sex differences. Moreover, differences in body fat distribution, such as the typical apple-shaped abdominal obesity in men and the pear-shaped gluteal obesity in women contribute to sex-specific cardiometabolic health susceptibilities [3, 30]. This is because sex differences in adipose tissue distribution with a predominance of subcutaneous fat depots in women and visceral fat in men result in different gene expression signatures and physiologic responses, such as adipokine production, adipogenic potential, the ability to store and mobilize lipids, and the interaction with the liver to deliver free fatty acids via the portal vein [10, 31]. To add to this complexity, several lifestyle factors likely affect the amount and composition of unhealthy visceral adipose tissue [32].

We found that several cardiometabolic risk factors were different between obese men and women despite accounting for the natural sex differences in body fat distribution and body fat percentages. This specifically includes the blood lipid levels HDL cholesterol and Apo A1 as well as triglycerides; parameters of glucose/insulin metabolism, such as fasting glucose and insulin levels, and its derived HOMA-IR relation, traditional liver enzymes ALAT and yGT, adiponectin as well as sE-selectin. Despite adjusting for body composition variables, significant differences remained for the above-mentioned parameters implying that these parameters are likely influenced by additional independent factors, e.g., sex hormones as oestrogens or genetic factors, and not alone by body composition differences between men and women.

In fact, clinical and experimental studies have demonstrated that sex steroid hormones influence sex differences in diabetes risk before menopause [33–35]. Later, menopause leads to an increase in the incidence of metabolic disorders [36, 37], but can be controlled at a low level with oestrogen-based replacement therapy (reviewed in [10]).

In general, in men and women the frequency of the MetS rises with increasing age with a peak at 60–69 years [36]. Notably, in our study cohort of predominantly very obese persons intending to start a medical weight loss program, the MetS was highly prevalent in young obese men < 40 years of age (MetS frequency 72.5%), but not in women (36.8%). Thus, in this age group the difference in MetS frequency between men and women was particularly evident, supporting the important role of female sex hormones in the protection of pre-menopausal women from metabolic and cardiovascular diseases [38].

However, we also found parameters that were seemingly sex different in univariate analysis, such as leptin levels, hsCRP levels and homocysteine levels, but where differences did not maintain after multivariate adjustments, including parameters of body composition. This implies, that these parameters represent surrogate indicators correlating with the amount of body fat and overall adiposity, as has been reported earlier [39–41].

The typical female body composition with lower waist circumference and other indices of less visceral fat depots, such as lower epicardial fat thickness, but higher body fat, and higher fat mass/lean mass-ratio could be affirmed in our study cohort. Thus, the importance of sex-specific differences in body composition emphasizing the concept of abdominal/ visceral adiposity for the emergence of many features of the MetS could be clearly supported by our data. In those few studies, that reported an apparently higher prevalence of the MetS in women than in men [42], the MetS was mostly combined with existing abdominal obesity in study cohorts of US [43], Indian [44] and Chinese [45] adults.

Therefore, an altered and increased ectopic fat content around the liver and visceral organs in men [7, 8, 46] might be a more potent determinant of metabolic health as an increased body fat mass by itself [3, 47].

The liver (and its physiological function in the regulation of energy storage and metabolic fluxes) is known to be a sexually dimorphic organ in terms of lipoprotein production and metabolic adaptions [48, 49]. Women, in turn, who are less susceptible to ectopic fat deposition in most tissues, such as the liver, are often protected from non-alcoholic fatty liver disease before menopause, which underlines once again a protective role of oestrogens [50]. Although, we clearly reported a sex difference in terms of different liver enzymes, we neither performed standardized abdominal and liver ultrasound nor elastography, limiting the validity of our results on sexually dimorphic metabolic liver disorders.

Central obesity and ectopic intraabdominal fat accumulation might also contribute to the sex differences in glucose and insulin metabolism. Our results are consistent with a large amount of literature showing that women are less prone to insulin resistance and metabolic dyslipidemia [51–53]. Especially in middle-aged populations prevalence of diabetes is more prevalent in men than in women in most parts of the word [51, 52]. Sex steroid hormones largely contribute to a better insulin sensitivity in women despite an increased fat mass and a lower skeletal muscle mass [54], implying that oestrogens confer protection against insulin resistance [10].

It has been also shown that sex has an impact on pancreatic endocrine function in multiple ways: women exhibit a greater insulin secretion capacity than men [54]; endogenous oestrogens exert protective effects on islets preserving beta cell function and preventing them from oxidative stress and lipotoxicity [55], and women secrete more glucose-dependent glucagon-like peptide-1 (GLP-1) following an oral glucose load [56].

Besides biological sex many gender-related behaviors exist that likely contribute to risk exposure [57, 58]. In our study cohort, women had a healthier lifestyle as compared to men. Specifically, alcohol consumption was less common in women than in men and in contrast to obese men, women reported a daily fruit and vegetable intake. Interestingly, some studies found that control of cardiovascular risk factors such as hypertension, and diabetes was better predicted by gender than by biological sex [59, 60]. Thus, we performed a detailed characterization and phenotyping, which allowed us comprehensive adjustments for various confounders that potentially influence sex effects on the prevalence of the MetS, which is our analysis' strength.

Thereby age, alcohol consumption, physical activity, and fruit and vegetable intake were additionally considered in the statistical models.

Adipose tissue as an endocrine organ produces a wide range of mediators regulating distinct pathways in the crosstalk between liver, skeletal muscle, pancreas and the cardiovascular system [5]. The mode of action of many adipokines released by adipose tissue cells is still largely unknown.

Adiponectin was found to be potentially protective in women irrespective of the obesity status in our study cohort. After adjustment for age, height, and %fat mass, we found sex-differences with higher expression of adiponectin in women than in men. Our results are in line with several studies, which reported sex-differences in adiponectin levels in non-obese persons [61–63]. However, we can demonstrate for the first time after a comprehensive adjustment strategy including body composition parameters that adiponectin is an important effect modifier to the sex-specific risk of the MetS.

Our study has several limitations. The study design of an observational cross-sectional study could not report a causality between sex differences in the MetS and adiponectin levels or abdominal adiposity. In various statistical adjustment models, we accounted for various confounders that potentially influence sex effects in the prevalence of the MetS, which is our analysis' strength. Age, body height, alcohol consumption, and fruit and vegetable intake and surrogate parameters of adiposity such as waist circumference were included in the models. Moreover, we cannot ensure that our study is powered enough to detect differences in all the variables described.

Abdominal adiposity was estimated by waist circumference and epicardial fat thickness as feasible and valid parameters for visceral fat [8]. We did not perform CT- or MRI-Scans or use the dual energy X-ray absorptiometry to evaluate the amount of visceral fat or body composition in detail. Neither abdominal ultrasound nor elastography were performed to assess fatty liver disease.

We recorded a detailed medical history and evaluation of cardiovascular diseases as well as thromboembolic events, but we didn’t assess comprehensive gynecological or endocrine diseases reflecting hormonal disturbances. There was no significant difference in parity between obese and non-obese women. In detail 70 obese and 22 non-obese women reported a previous pregnancy (*p* = 0.514, data not shown). Three obese and one non-obese women developed preeclampsia. Abortion was numerically but not significantly higher in obese woman compared to non-obese (*n* = 22 vs. *n* = 3, data not shown). We cannot provide other detailed obstetric complications.

Moreover, we have not measured plasma levels of sex hormones and were thus not able to exactly analyze their effects on the MetS prevalence. We also did not survey the menopausal status. However, determining menopausal status is not trivial in epidemiological studies, because the transition from pre-menopause to post-menopause often lasts several years and varies in duration and symptomology. Moreover, there are still no standard definitions to define menopausal status using observational data.

## Conclusions

Beyond the degree of adiposity considerable sex differences with regard to body fat distribution may potentially contribute to the prevalence of the MetS and, ultimately cardiovascular disease. It is essential to understand the impact of sex, abdominal adiposity, and adipocytokines on obesity and cardiometabolic diseases. Our study results confirm that women had a more favourable metabolic profile than men despite a higher fatmass/lean mass ratio. In several statistical regression models adjusting for various potential confounding factors, such as lifestyle and body composition, we show that parameters of visceral (unhealthy) adiposity such as waist circumference largely contribute to the sex differences in the prevalence of the MetS independent of the amount of adiposity. Moreover, additional influences of adipokines such as adiponectin, and sex hormones likely contribute to the lower susceptibility to the MetS in women compared to men.

## Supplementary Information

**Additional file 1: Table S1:** Proportions of lifestyle factors in non-obese and obese study participants. **Table S2:** Unadjusted means of lipid, metabolic and inflammatory markers and their standard deviations in non-obese and in grade 1 to grade 3 obese study participants. **Table S3:** Unadjusted means of cardiovascular parameters and their standard deviations in non-obese and in grade 1 to grade 3 obese study participants.

## Data Availability

The data sets generated and/or analyzed during the current study are not publicly available due to ongoing studies but are available from the corresponding author on reasonable request.
