# Supplementary material for: Gender differences in cardiometabolic health and disease in a cross-sectional observational obesity study
Source: Biol Sex Differ. 2022 Mar 4;13:8. doi: 10.1186/s13293-022-00416-4 (PMC8897897; doi:10.1186/s13293-022-00416-4)
Supplement: Supplementary file 1 — Additional file 1: Table S1: Proportions of lifestyle factors in non-obese and obese study participants. Table S2: Unadjusted means of lipid, metabolic and inflammatory markers and their standard deviations in non-obese and in grade 1 to grade 3 obese study participants. Table S3: Unadjusted means of cardiovascular parameters and their standard deviations in non-obese and in grade 1 to grade 3 obese study participants. [file 13293_2022_416_MOESM1_ESM.docx]

**Additional file 1: Table S1:** Proportions of lifestyle factors in non-obese and obese study participants.

|  | Non-Obese  female | male | p-value | Obese female | male | p-value |
| --- | --- | --- | --- | --- | --- | --- |
| *Smoking* |  |  |  |  |  |  |
| Never [n, (%)] | 26 (54.2%) | 21 (77.8%) |  | 109 (52.2%) | 58 (40.6%) |  |
| Former [n, (%)] | 14 (29.2%) | 4 (14.8%) | 0.149 | 70 (33.5%) | 60 (42.0%) | 0.103 |
| Current [n, (%)] | 8 (16.7%) | 2 (7.4%) |  | 30 (14.4%) | 25 (17.5%) |  |
| *Alcohol consumption* |  |  |  |  |  |  |
| Non or rarely [n, (%)] | 36 (75.0%) | 13 (52.0%) | 0.066 | 179 (87.3%) | 80 (56.3%) | <0.0001 |
| Regularly or daily [n, (%)] | 12 (25.0%) | 12 (48.0%) |  | 26 (12.7%) | 62 (43.7%) |  |
| *Fruits & vegetables intake* |  |  |  |  |  |  |
| 0-3 x per week [n, (%)] | 8 (16.7%) | 8 (32.0%) | 0.115 | 63 (31.0%) | 76 (55.5%) | <0.0001 |
| Daily [n, (%)] | 40 (83.3%) | 17 (68.0%) |  | 140 (69.0%) | 61 (44.5%) |  |
| *Physical exercise* |  |  |  |  |  |  |
| 0-2 x per week [n, (%)] | 17 (36.2%) | 10 (40.0%) | 0.801 | 112 (54.6%) | 89 (62.7%) | 0.151 |
| ≥3 x per week [n, (%)] | 30 (63.8%) | 15 (60.0%) |  | 93 (45.4%) | 53 (37.3%) |  |

Lifestyle characteristics of study cohort stratified by sex and obesity status. In non-obese and obese study participants smoking behaviour and physical activity was similar in men and women; alcohol consumption was significantly higher in men; the consumption of fruits and vegetables was significantly lower in men than in women. Data are shown as numbers and percentages. P-values derived from standard two-way tables. The Pearson χ2 test was used to calculate for the independence of the rows and columns.

**Additional file 1: Table S2:** Unadjusted means of lipid, metabolic and inflammatory markers and their standard deviations in non-obese and in grade 1 to grade 3 obese study participants.

|  | Non-Obese | Grade 1 Obese | Grade 2 Obese | Grade 3 Obese | p-value  female vs. male |
| --- | --- | --- | --- | --- | --- |
| ***Lipids*** |  |  |  |  |  |
| Total-Chol [mg/dL] |  |  |  |  |  |
| female | 210±42 | 192±31 | 206±32 | 204±36 |  |
| male | 193±33 | 204±43 | 198±40 | 193±38 | n.s. |
| LDL-Chol [mg/dL] |  |  |  |  |  |
| female | 120±30 | 115±29 | 127±27 | 125±30 |  |
| male | 113±32 | 130±37 | 126±33 | 126±36 | n.s. |
| HDL-Chol [mg/dL] |  |  |  |  |  |
| female | 68±19 | 56±13 | 55±15 | 53±13 |  |
| male | 59±11 | 41±8 | 41±8 | 41±9 | <0.0001 |
| Triglycerides [mg/dL] |  |  |  |  |  |
| female | 103±63 | 110±50 | 127±54 | 144±97 |  |
| male | 103±67 | 176±93 | 183±96 | 165±79 | <0.0001 |
| Lp(a) [mg/dL] |  |  |  |  |  |
| female | 33±24 | 29±19 | 34±28 | 36±29 |  |
| male | 45±36 | 34±23 | 31±22 | 36±30 | n.s. |
| oxLDL [mg/dL] |  |  |  |  |  |
| female | 51±20 | 54±23 | 56±22 | 56±24 |  |
| male | 48±19 | 58±38 | 56±21 | 60±25 | n.s. |
| ApoB-48 [µg/dL] |  |  |  |  |  |
| female | 7.4±4.0 | 6.9±4.4 | 8.5±3.6 | 6.7±4.7 |  |
| male | 9.5±4.4 | 10.6±4.5 | 9.0±3.6 | 9.6±4.7 | n.s. |
| ApoA-1 [mg/dL] |  |  |  |  |  |
| female | 185±32 | 159±27 | 164±33 | 164±33 |  |
| male | 167±23 | 140±15 | 140±19 | 140±21 | <0.0001 |
| ApoA-2 [mg/dL] |  |  |  |  |  |
| female | 38±8 | 33±7 | 33±6 | 34±7 |  |
| male | 35±5 | 33±5 | 34±6 | 32±8 | n.s. |
| ApoB [mg/dL] |  |  |  |  |  |
| female | 94±25 | 93±20 | 102±20 | 100±23 |  |
| male | 86±23 | 107±23 | 105±22 | 106±23 | n.s. |
| PCSK9 [ng/mL] |  |  |  |  |  |
| female | 12.2±6.0 | 15.7±4.9 | 16.2±4.8 | 16.7±3.9 |  |
| male | 11.0±2.7 | 14.0±4.0 | 15.3±4.5 | 15.7±6.3 | n.s. |
| ***Insulin-Glucose*** |  |  |  |  |  |
| Glucose [mg/dL] |  |  |  |  |  |
| female | 85±8 | 88±11 | 98±25 | 96±19 |  |
| male | 86±9 | 99±17 | 103±34 | 118±45 | 0.0003 |
| Insulin [mU/L] |  |  |  |  |  |
| female | 8.8±5.9 | 11.5±5.6 | 18.8±12.7 | 26.2±25.4 |  |
| male | 6.6±3.7 | 14.7±7.6 | 22.4±10.9 | 39.1±24.5 | 0.012 |
| HOMA-IR |  |  |  |  |  |
| female | 1.9±1.4 | 2.6±1.4 | 5.1±5.5 | 6.4±6.2 |  |
| male | 1.4±0.9 | 3.6±1.8 | 6.2±5.2 | 11.7±10.7 | 0.0042 |
| ***Liver enzymes*** |  |  |  |  |  |
| GPT(ALAT) [mg/dL] |  |  |  |  |  |
| female | 25±13 | 29±15 | 34±22 | 31±18 |  |
| male | 30±11 | 43±23 | 52±32 | 51±39 | <0.0001 |
| GOT (ASAT) [mg/dL] |  |  |  |  |  |
| female | 22±8 | 21±8 | 24±12 | 21±10 |  |
| male | 26±11 | 26±11 | 28±11 | 29±18 | n.s. |
| yGT [mg/dL] |  |  |  |  |  |
| female | 26±24 | 26±13 | 32±20 | 37±28 |  |
| male | 40±61 | 45±24 | 59±39 | 56±35 | <0.0001 |
| Alkaline phosphatase [mg/dL] |  |  |  |  |  |
| female | 65±19 | 71±21 | 77±17 | 80±10 |  |
| male | 72±17 | 66±21 | 75±23 | 80±20 | n.s. |
| ***Other biomarkers*** |  |  |  |  |  |
| Leptin [µg/L] |  |  |  |  |  |
| female | 19.4±13.4 | 47.5±52.2 | 61.0±22.8 | 91.3±65.9 |  |
| male | 3.3±1.7 | 11.0±6.7 | 23.6±14.2 | 50.9±23.1 | <0.0001 |
| Adiponectin [µg/mL] |  |  |  |  |  |
| female | 12.2±5.2 | 9.8±3.8 | 9.6±3.1 | 10.7±4.9 |  |
| male | 10.4±9.3 | 6.8±2.5 | 6.9±2.8 | 7.4±3.0 | <0.0001 |
| Resistin [ng/mL] |  |  |  |  |  |
| female | 5.7±3.1 | 6.8±5.1 | 6.8±4.0 | 7.9±5.5 |  |
| male | 6.0±2.5 | 5.3±2.3 | 6.0±4.1 | 6.8±4.3 | n.s. |
| FABP4 [ng/mL] |  |  |  |  |  |
| female | 22±13 | 34±15 | 38±13 | 47±15 |  |
| male | 17±9 | 27±12 | 34±10 | 48±16 | n.s. |
| Activin A [pg/mL] |  |  |  |  |  |
| female | 303±108 | 328±87 | 362±136 | 385±139 |  |
| male | 296±60 | 335±93 | 351±98 | 397±158 | n.s. |
| TNFalpha [pg/mL] |  |  |  |  |  |
| female | 8.7±2.9 | 9.0±4.4 | 9.2±3.5 | 9.4±2.8 |  |
| male | 9.7±4.4 | 9.1±2.9 | 9.5±2.5 | 10.9±4.7 | n.s. |
| hsCRP [mg/L] |  |  |  |  |  |
| female | 1.7±1.8 | 4.5±5.6 | 8.1±10.1 | 10.9±10.5 |  |
| male | 1.4±2.1 | 2.5±3.0 | 4.0±3.8 | 7.7±5.4 | <0.0001 |
| IL-6 [pg/mL] |  |  |  |  |  |
| female | 3.6±1.6 | 4.8±5.1 | 3.4±0.9 | 5.1±2.9 |  |
| male | 2.9±0.5 | 4.0±1.9 | 3.7±1.4 | 4.9±2.2 | n.s. |
| Homocystein [µmol/L] |  |  |  |  |  |
| female | 9.5±2.3 | 10.0±3.0 | 10.3±2.8 | 10.0±3.2 |  |
| male | 11.0±2.4 | 12.6±9.4 | 12.6±5.0 | 11.2±3.6 | <0.0001 |
| MMP-9 [ng/mL] |  |  |  |  |  |
| female | 484±206 | 572±272 | 528±204 | 750±397 |  |
| male | 583±271 | 465±175 | 608±236 | 719±352 | n.s. |
| CD40L [ng/mL] |  |  |  |  |  |
| female | 7187±2164 | 7621±3009 | 8588±2863 | 7621±3102 |  |
| male | 8887±3733 | 5933±2718 | 7130±3296 | 9184±3479 | n.s. |
| sE-Selectin [ng/mL] |  |  |  |  |  |
| female | 33±14 | 37±23 | 47±30 | 46±20 |  |
| male | 39±14 | 40±18 | 50±18 | 55±28 | 0.0292 |
| sICAM [ng/mL] |  |  |  |  |  |
| female | 216±74 | 225±61 | 243±82 | 260±97 |  |
| male | 206±61 | 213±85 | 247±76 | 286±107 | n.s. |
| sVCAM [ng/mL] |  |  |  |  |  |
| female | 678±204 | 609±157 | 604±167 | 623±199 |  |
| male | 711±289 | 591±233 | 636±311 | 692±279 | n.s. |

Parameters of blood lipids, insulin-glucose metabolism, liver enzymes, adipokines, and markers of inflammation and early atherogenesis were studied for sex differences across increasing obesity severity grades. P Values refer to an overall test to assess whether there is a difference between men and women among all obesity grades.

**Additional file 1: Table S3:** Unadjusted means of cardiovascular parameters and their standard deviations in non-obese and in grade 1 to grade 3 obese study participants.

|  | Non-Obese | Grade 1 Obese | Grade 2 Obese | Grade 3 Obese | p-value  female vs. male |
| --- | --- | --- | --- | --- | --- |
| Systolic BP [mmHg] |  |  |  |  |  |
| female | 126±16 | 130±13 | 137±15 | 143±20 |  |
| male | 130±13 | 139±14 | 142±14 | 146±17 | <0.0001 |
| Diastolic BP [mmHg] |  |  |  |  |  |
| female | 80±11 | 83±9 | 89±12 | 93±12 |  |
| male | 81±8 | 88±8 | 89±10 | 89±15 | n.s. |
| Pulse Pressure [mmHg] |  |  |  |  |  |
| female | 46±9 | 47±10 | 48±10 | 49±13 |  |
| male | 50±8 | 51±11 | 53±10 | 57±11 | 0.0025 |
| Heart Rate [beats/min] |  |  |  |  |  |
| female | 70±14 | 70±11 | 74±13 | 75±13 |  |
| male | 65±13 | 64±10 | 69±12 | 76±15 | <0.0001 |
| IMT [mm] |  |  |  |  |  |
| female | 0.57±0.18 | 0.64±0.19 | 0.65±0.13 | 0.68±0.18 |  |
| male | 0.54±0.17 | 0.73±0.17 | 0.72±0.25 | 0.76±0.21 | <0.0001 |
| Ankle/Brachial-Index |  |  |  |  |  |
| female | 1.02±0.16 | 1.07±0.17 | 1.05±0.17 | 1.04±0.16 |  |
| male | 1.02±0.12 | 1.09±0.14 | 1.07±0.12 | 1.02±0.19 | n.s. |
| NTproBNP [pg/mL] |  |  |  |  |  |
| female | 84±82 | 77±65 | 78±101 | 93±86 |  |
| male | 31±18 | 49±51 | 62±110 | 107±402 | n.s. |
| e’ velocity [cm/s] |  |  |  |  |  |
| female | 15.4±6.9 | 13.5±6.1 | 13.0±6.5 | 12.8±4.7 |  |
| male | 16.5±6.5 | 10.1±3.1 | 12.3±4.0 | 11.9±3.4 | <0.0001 |
| E/e’ |  |  |  |  |  |
| female | 6.4±2.5 | 6.7±2.4 | 6.4±2.0 | 7.4±2.7 |  |
| male | 5.3±1.4 | 7.2±2.4 | 6.9±2.7 | 7.2±2.4 | n.s. |
| LA area [cm2] |  |  |  |  |  |
| female | 15.4±3.0 | 17.3±2.3 | 18.4±4.5 | 13.6±3.5 |  |
| male | 16.1±4.0 | 19.7±3.2 | 21.4±5.1 | 23.8±7.7 | <0.0001 |
| LV mass [g] |  |  |  |  |  |
| female | 94±18 | 105±23 | 106±22 | 112±25 |  |
| male | 118±27 | 135±31 | 137±30 | 135±27 | 0.0001 |
| Epicardial fat thickness [mm] |  |  |  |  |  |
| female | 2.6±2.4 | 5.1±2.6 | 5.8±2.2 | 7.3±2.8 |  |
| male | 1.8±1.7 | 6.3±2.5 | 7.1±2.9 | 8.7±3.7 | <0.0001 |
| Ejection fraction [%] |  |  |  |  |  |
| female | 67±7 | 65±6 | 65±8 | 65±6 |  |
| male | 71±8 | 64±8 | 63±6 | 62±7 | n.s. |

Cardiovascular parameters (blood pressure parameters, intima media thickness, ankle/brachial index as well as echocardiographic parameters) were studied for sex differences across increasing obesity severity grades. P Values refer to an overall test to assess whether there is a difference between men and women among all obesity grades.
